# Structural cycle of the *Thermus thermophilus* PilF ATPase: the powering of type IVa pilus assembly

**DOI:** 10.1038/s41598-018-32218-3

**Published:** 2018-09-19

**Authors:** Richard Collins, Vijaykumar Karuppiah, C. Alistair Siebert, Rana Dajani, Angela Thistlethwaite, Jeremy P. Derrick

**Affiliations:** 1 School of Biological Sciences, Faculty of Biology, Medicine and Health, Manchester Academic Health Science Centre, The University of Manchester, Oxford Road, Manchester, UK; 2 Electron Bio-Imaging Centre, Diamond Light Source, Harwell Science & Innovation Campus, Didcot, Oxfordshire UK

## Abstract

Type IV pili are responsible for a diverse range of functions, including twitching motility and cell adhesion. Assembly of the pilus fiber is driven by a cytoplasmic ATPase: it interacts with an inner membrane complex of biogenesis proteins which, in turn, bind to nascent pilin subunits and mediate fiber assembly. Here we report the structural characterization of the PilF TFP assembly ATPase from *Thermus thermophilus*. The crystal structure of a recombinant C-terminal fragment of PilF revealed bound, unhydrolysed ATP, although the full length complex was enzymatically active. 3D reconstructions were carried out by single particle cryoelectron microscopy for full length apoprotein PilF and in complex with AMPPNP. The structure forms an hourglass-like shape, with the ATPase domains in one half and the N1 domains in the second half which, we propose, interact with the other pilus biogenesis components. Molecular models for both forms were generated: binding of AMPPNP causes an upward shift of the N1 domains towards the ATPase domains of ~8 Å. We advocate a model in which ATP hydrolysis is linked to displacement of the N1 domains which is associated with lifting pilin subunits out of the inner membrane, and provide the activation energy needed to form the pilus fiber.

## Introduction

Type IV pili (TFP) are widespread in Gram negative bacteria, including several notable human pathogens^1,2^. Expression of TFP is linked to a variety of important functions, including adhesion to epithelial cells^3^, DNA uptake^4^ and twitching motility, which is responsible for motion across solid surfaces^5^. Each pilus fiber consists principally of a non-covalent polymer of pilin protein subunits, arranged with helical symmetry^6^. TFP are built up by addition of pilin subunits from the base of the fiber. Electron microscopy and computational analysis have provided evidence that the pilus fiber is built by addition of one pilin subunit at a time, in a rotatory mechanism^7^. Pilins from all sources studied to date share a highly hydrophobic N-terminal α-helix which allows them to remain in the inner membrane in an unpolymerised state until they are recruited into a nascent pilus fiber^6^.

Formation of TFP is promoted by a complex of specialist biogenesis proteins which span the inner and outer membranes^1^. The pilus fiber crosses the outer membrane by passage through a secretin, an integral membrane protein which forms a channel to accommodate the assembled fiber^8^. Fiber assembly occurs in the periplasm, probably close to the inner membrane, although the molecular details of this process are obscure. Three proteins, PilM, PilN and PilO, form an inner membrane platform and play a critical role in this process. PilM is a soluble protein structurally similar to the actin-like protein FtsA^9^; it binds ATP and also a short N-terminal cytoplasmic segment of PilN, which is a monotopic integral membrane protein. PilM and PilN bind to PilO, also a monotopic integral membrane protein^10,11^. The PilMNO complex binds to PilP, a lipoprotein which mediates binding to the PilQ secretin in the outer membrane^8,12^. Recent evidence has shown that *N. meningitidis* PilMNOP can be expressed in *E. coli* and isolated as a stable complex^13^.

A third vital component of the TFP biogenesis system is the assembly ATPase (PilF in *T. thermophilus*). The TFP assembly ATPases are part of the extensive AAA+ superfamily which form molecular motors, using the energy from ATP hydrolysis to drive mechanical transformations^14^. Within this superfamily, PilF shares a number of sequence similarities, in terms of its domain organisation, with GspE, an ATPase which provides the energy for type II secretion. Other TFP biogenesis proteins also have counterparts in the type II secretion system (T2SS), including the outer membrane secretin and the PilMNO inner membrane platform^1,2^. These parallels are suggestive of a similar fundamental mechanism operating in the two secretion systems. One model for the operation of the T2SS is through the assembly/disassembly of pseudopilins, which are proposed to participate in a ‘piston-shunt’ type mechanism, driving passage of the secreted product through the secretin to the exterior^15^. A compelling line of evidence for the similarities between the two systems lies in the observation that a T2SS can be used to assemble a TFP fiber^16^.

The domain structure of T2SS and TFP biogenesis ATPases is similar, in that they comprise one or more N1 or GSPII domains at the N-terminus, followed by a C-terminal (CTD) and N2 domain- which comprise the ATP binding site. The CTD and N2 domains are highly conserved, at a structural level, within the AAA+ superfamily^14^. Recent crystal structures of PilF from *T. thermophilus* and PilB from *Geobacter metallireducens* have shown how hydrolysis could be linked to structural changes in the relative positions of the CTD and N2 domains^17,18^. Critically, however, the N1 domains were absent or not resolved in these structures. The results are consistent with studies carried out on related ATPases which indicate that the relative orientations between the two domains vary widely^19^.

TFP are also capable of rapid disassembly, a process which generates powerful motor forces responsible for twitching motility and is energised by the PilT ATPase^5^. PilT ATPases share some of the domain organisation of the GspE/PilF family and they also harbour N2 and CTD domains, but lack a Zn-binding motif and the N1 domains. Nevertheless, their structural organisation appears to be similar, in that they form hexamers, and the relative motions of the N2 and CTD domains are apparently linked to ATP hydrolysis^20^. The emerging picture is therefore that these two groups of ATPases function in a broadly similar way. What is much less clear, however, is how mechanical displacement of the N2 domain is linked to pseudopilus/TFP assembly or disassembly.

Recent reconstructions of the entire TFP assembly machinery by cryoelectron tomography in *T. thermophilus*^21^, *Myxococcus xanthus*^22^ and *Vibrio cholerae*^23^ show a complex which spans the inner and outer membranes, providing direct evidence that cytoplasmic ATPases are linked, through the inner membrane platform complex, to pilus assembly. Previously we have shown that pilin monomer binds to the *Thermus* PilMNO complex *in vitro*, suggesting that it plays a role in transducing ATP-driven mechanical force across the inner membrane^10^. Here we present a combination of X-ray crystallographic and cryoelectron microscopy single particle reconstructions of the PilF hexamer in two states which are used to build molecular models for the complete PilF hexamer and chart its structural transition on binding of ATP. These observations therefore complete a critical link in the type IV pilus assembly pathway, and allow us to present a structural model for the transmission of mechanical displacement of the N2 domain, through to the N1 domains and, by implication, PilMNO and bound pilin.

## Results

### Crystal structure of the C-terminal portion of the TtPilF assembly ATPase

Crystallization trials on the complete TtPilF hexamer were unsuccessful; however, limited proteolysis gave rise to a fragment of approximate molecular mass 44 kDa which was purified and yielded crystals which diffracted to 2.44 Å (Supplementary Table S1). Mass spectrometry was used to confirm that this fragment corresponded to the C-terminal half of the protein, encompassing the N2, CTD and Zn-binding domains (designated TtPilF_c_). The structure was solved using phases obtained from SeMet-labelled protein (Supplementary Table S1), which was necessary to assist in a molecular replacement solution of the structure.

**Figure 1:**
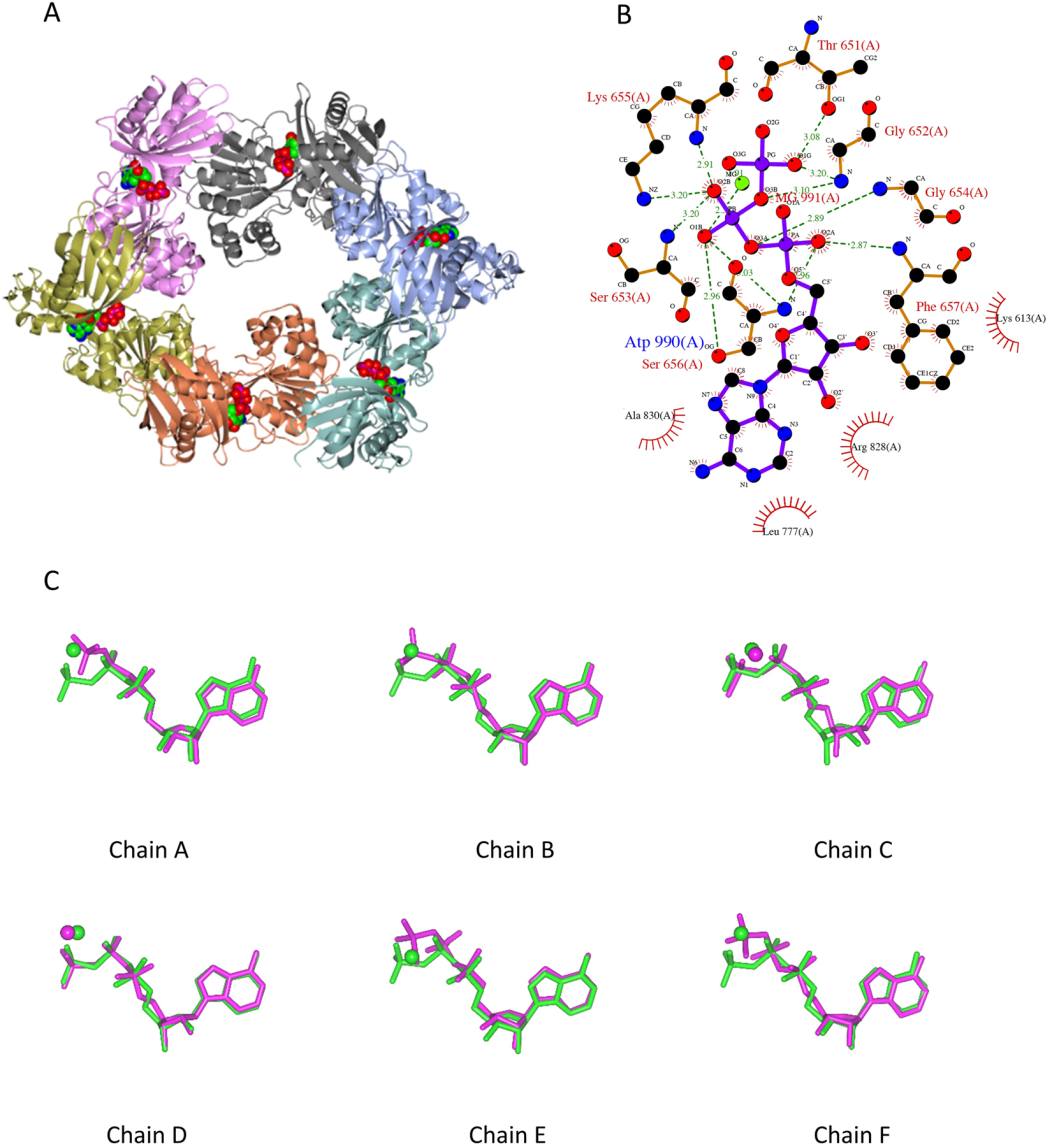
Structure of the TtPilF_c_ hexamer with comparison of binding of ATP and ATPγS. (**A**) Ribbon plot of TtPilF_c_ with ATP molecules shown in spacefilling form. (**B**) ATP binding site visualised using LigPlot^49^. Example shown is for chain A. (**C**) Comparison of the conformations of ATP/Mg^2+^ and ATPγS in the two different TtPilF_c_ crystal forms. Panels are labelled by chain number, as specified in the ATP/Mg^2+^ complex. Each chain was superimposed on its counterpart, as implemented within CCP4MG^48^.

TtPilF_c_ forms a flattened hexamer, with 2-fold rotational non-crystallographic symmetry relating the two trimers (Fig. 1A). TtPilF_c_ is identical in sequence but in a different crystal form to a structure published previously for a similar TtPilF fragment derived from a different *Thermus thermophilus* strain^18^. An alignment of both structures gave an r.m.s.d. of 0.354 Å; an overlay of TtPilF_c_ with PilB from *Geobacter metallireducens*, which also has 2-fold rotational symmetry within the hexamer, gave an r.m.s.d. of 1.564 Å (Supplementary Fig. S1A,B). Although the two forms of TtPilF_c_ crystallised in different space groups, their crystal packing was very similar (Supplementary Fig. S1C,D). Inspection of all 6 binding sites revealed electron density consistent with bound nucleotide; however, there was no evidence for ATP hydrolysis in any site (Supplementary Fig. S2). ATPase assays were carried out on the TtPilF_c_ fragment which showed that it was not enzymatically active, confirming that the fragment is catalytically inert in solution, as well as in the crystalline state. Superposition of all chains showed that the conformation of ATP/Mg^2+^ in the 6 binding sites is similar (Supplementary Fig. S3). This observation shows that the ATP conformation is unaffected by differences in contacts between adjacent subunits caused by flattening of the hexamer (Fig. 1A). Recognition of bound ATP was similar for all chains- details of interacting residues are shown in Fig. 1B. Thr651, Gly652, Ser653, Lys655 and Ser656 form part of the Walker A motif involved in binding the γ and β phosphates. These residues, as expected, are highly conserved within the PilB family^17^.

The principal difference between the PilFc structure reported here and that presented by Mancl *et al*.^18^, is that the latter contained phosphothiophosphoric acid–adenylate ester (ATPγS), an ATP analog with S substituted for O in the γ phosphate. ATPγS is a substrate for most protein kinases, although the activity levels are probably considerably lower than for the natural ATP substrate^24^. The complex with ATPγS also gave no indication of hydrolysis of the terminal phosphate, in keeping with our observations. We systematically compared the conformations of the two ligands in both structures for each equivalent binding site (Fig. 1C). Mg^2+^ occupies the position of the thiophosphate moiety in chains A, B, E and F; for the remaining two chains, the conformation of both nucleotides is very similar, with Mg^2+^ ions bound to the ATPγS. Furthermore, where density for Mg^2+^ ions was present in the ATPγS complex, we noticed that it was weaker than the six copies in the ATP complex. We conclude that the variation in conformation of bound ATPγS between different sites is attributable to the weaker electronegativity of S compared with O, reducing the polarity of the S = P bond, compared with O = P and changing the local charge distribution.

One possible explanation for the failure of the TtPilF_c_ fragment to hydrolyse ATP is the conformation of the linker polypeptide between the CTD and N2 domains. A comparison of TtPilF_c_ chain A with equivalent subunits from *Geobacter metallireducens* PilB in ADP- and AMPPNP-bound forms showed significant deviations in the linker region (Supplementary Fig. S4). Small changes in the orientation of catalytic residues within the active site can have a dramatic effect on catalytic efficiency.

### Cryoelectron microscopy structures of TtPilF in the apoprotein and AMPPNP-bound states

Cryoelectron microscopy was used in conjunction with single particle averaging to generate 3D reconstructions of the complete TtPilF hexamer, as an apoprotein and in complex with AMPPNP (sample data are shown in Supplementary Fig. S5). We have previously reported the structures of TtPilF in the two states previously, but at lower resolution^25^. In our previous work we clearly identified the complex as a hexamer, but at lower resolution there was no evidence of a C2 symmetry deviation: we therefore refined the structures of both forms of TtPilF with C6 symmetry. However, it became apparent during the processing of the superior direct detector data that a subtle C2 symmetry was apparent in the complex top-view (see exemplar class averages in Supplementary Fig. S5C). Furthermore, a C2 symmetry designation would be consistent with the crystal structures of the ATPase domains discussed previously. This was obvious during the processing stages and application of C6 symmetry-produced maps, with good correspondence between class averages and structure back projections only for side views. Estimation of the resolution of the TtPilF map (AMPPNP-bound form) by FSC correlation gave a value of 8 Å at the conservative value of 0.5 (Supplementary Fig. S6A). Calculation of the map resolution by ResMap^26^ showed an even distribution of resolution through the height of the complex, and suggested a higher overall resolution value of 4–5 Å (Supplementary Fig. S6B,D). Visual inspection of the map suggested that the more conservative value from the FSC correlation is likely to be closer to the correct value. The same calculations carried out on the TtPilF apoprotein map gave a similar resolution estimate from the FSC value, although the ResMap estimate was slightly worse, at 5–6 Å.

**Figure 2:**
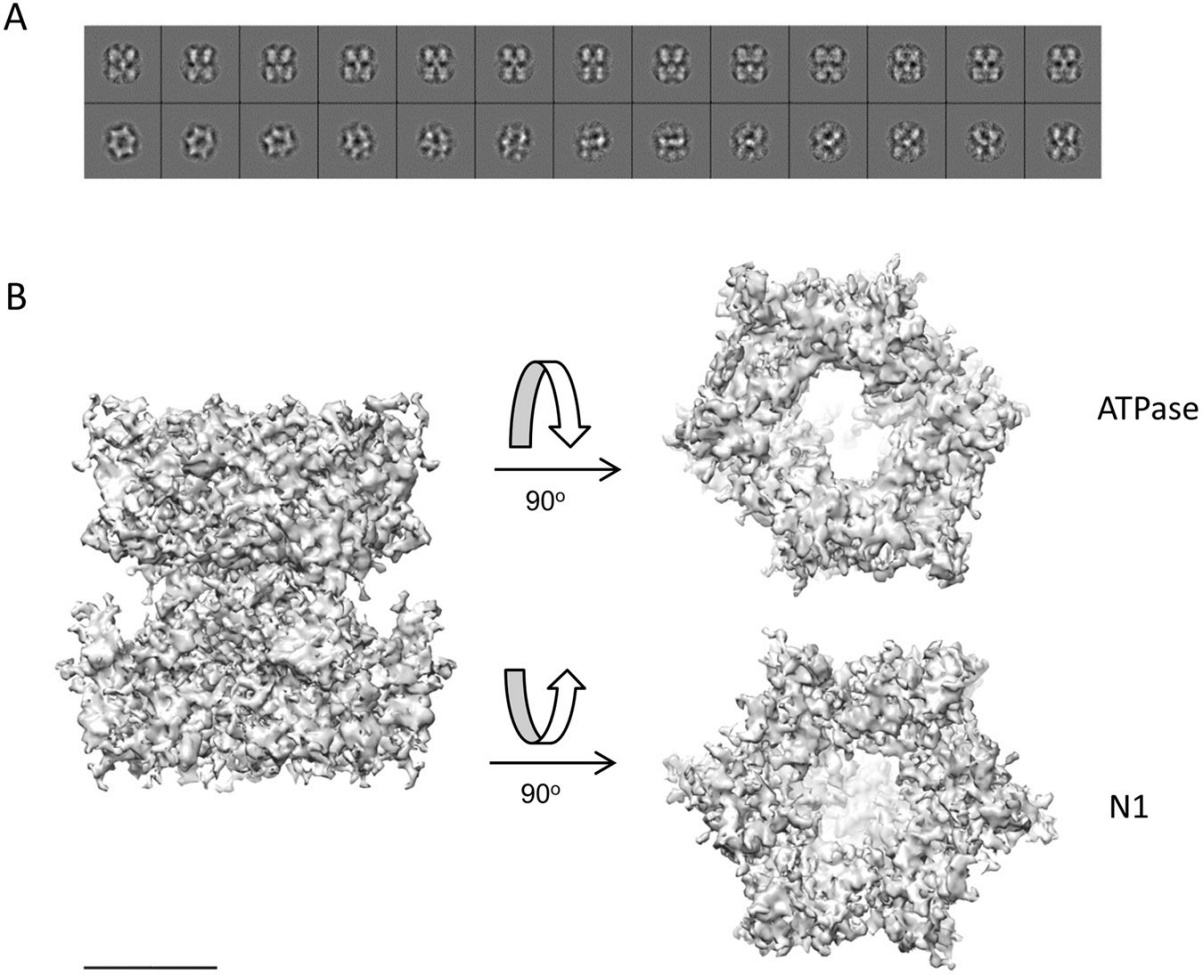
Cryoelectron microscopy structure of TtPilF in the AMPPNP-bound form. (**A**) A selection of high resolution unsymmetrized and reference free projection class averages showing a variety of Top to Side views from the dataset of 40,000 particles. These data indicate a good sampling of EULER space. Box size = 192 Å. (**B**) A surface render of the TtPilF density map; the map was normalised and extraneous non protein density noise reduced using the ‘Hide Dust’ option, as implemented within Chimera^33^. Side, top and bottom views are shown, such that the top view corresponds to the ATPase region and the bottom to the N1 domains region. Scale bar = 50 Å.

The electron density maps obtained were similar in their general features to those determined previously^25^, but with considerably more detail; an example of the map quality for the AMPPNP-bound state is shown in Fig. 2. The structure divided into two approximately equal masses although, at this improved resolution, it was readily apparent that they are different. There was also much more detail around the central region, which appears more complex than the simple ‘stem’ structure which was observed previously^25^. Of particular note is the fact that the part of the structure associated with the ATPase domains is more distorted from C6 symmetry than the N1 domain section (Fig. 2B). A comparison of the two density maps revealed that they are similar in overall shape and dimensions, but the apoprotein structure is shorter and wider than the AMPPNP structure, indicative of conformational change between the two states (discussed further below).

### Comparison of the structures of the ATPase domains

**Figure 3:**
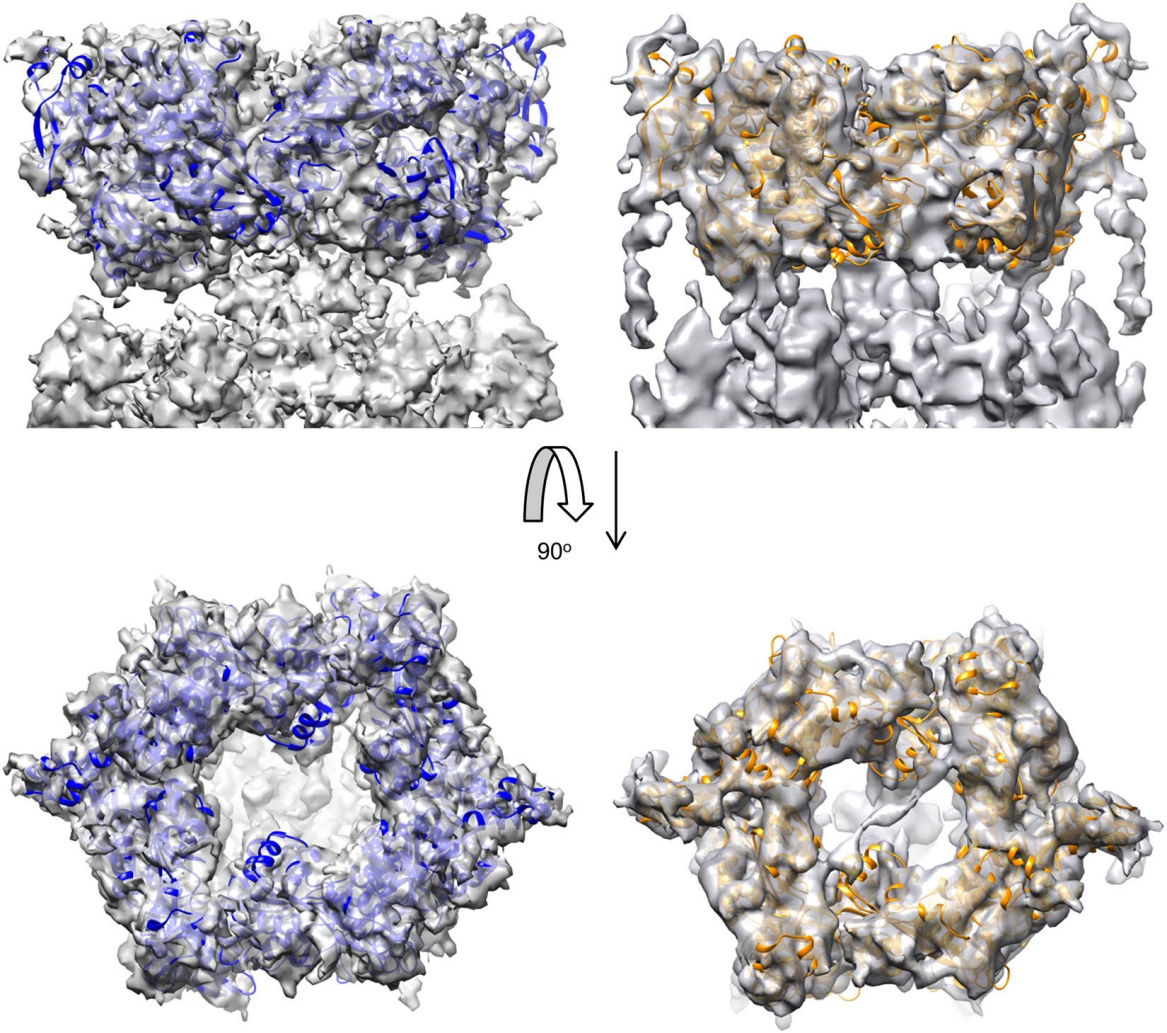
Docked conformations of TtPilF ATPase domains into cryoelectron density maps from the AMPPNP and apoprotein forms. Side and top views of ATPase domains are shown docked into the AMPPNP (left) and apoprotein (right) cryoelectron density maps. The AMPPNP-bound structure is in blue and the apoprotein is in orange. Domains from some chains have been omitted in the side views, for clarity.

To construct a molecular model for the TtPilF hexamer, we examined the fitting of the TtPilF_c_ crystal structure into the AMPPNP cryoelectron density map. Two alternative positions were possible which were evaluated independently by rigid body fitting (Supplementary Fig. S7). The correctly docked form was readily identified by a superior correlation coefficient (0.83 versus 0.70), which reflected a much better fit of the crystal structure into the top half of the density map (Supplementary Fig. S7). The fit of the docked model was optimized by refinement in ROSETTA^27^, giving the model TtPilF^AMPPNP^. The TtPilF^AMPPNP^ structure was then used as a starting point for refinement against the apoprotein map, and the final model was designated TtPilF^apo^; structures of both forms are shown in Fig. 3. Electron density was complete for almost all of both models in this region of the density map.

**Figure 4:**
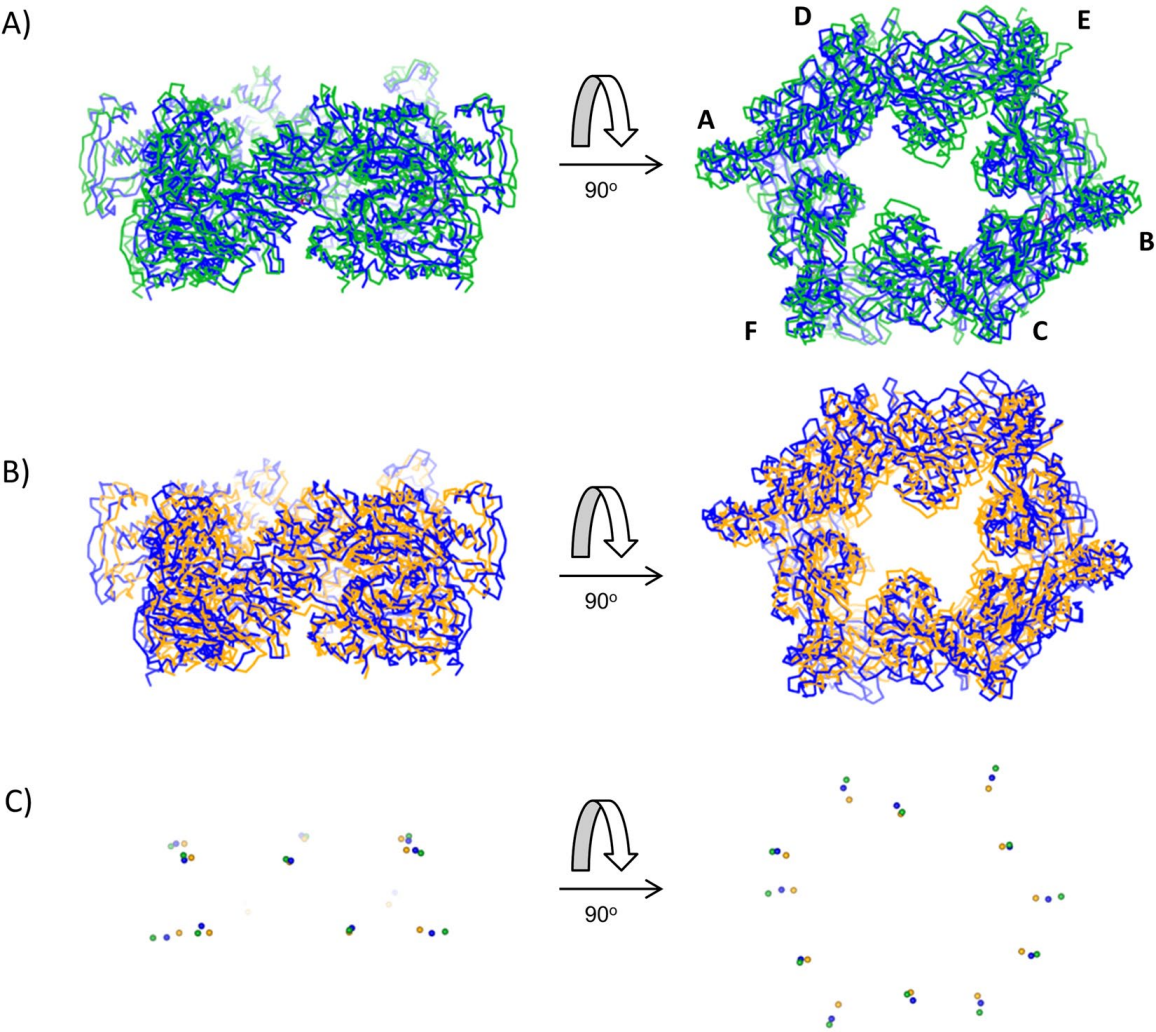
Comparison of structures and centers of mass of ATPase domains from PilF_c_, TtPilF^AMPPNP^ and TtPilF^apo^. (**A**) Orthogonal views of superimposed Cα traces of PilF_c_ (green) and TtPilF^AMPPNP^ (blue). (**B**) Orthogonal views of superimposed Cα traces of TtPilF^AMPPNP^ (blue) and TtPilF^apo^ (orange). The same orientations are used as in (**A**). (**C**) Centers of mass for each CTD and N2 domain, plotted as dummy atoms, for PilF_c_, TtPilF^AMPPNP^ and TtPilF^apo^. The same orientations and color conventions are used as (**A**,**B**).

Significant differences were apparent between the TtPilF^AMPPNP^ model and the TtPilF_c_ crystal structure. Superposition of the CTD and N2 domains gave an r.m.s. displacement of 3.47 Å for Cα atoms; when viewed from the top, TtPilF_c_ is wider by about 6 Å along the long axis and shorter by 7 Å along the shorter axis, leading to a flatter hexagonal shape (Fig. 4A). Given that the hexamer adopts C2 symmetry, three chains were compared (F, C and B) which lie on one side of the hexamer. Superposition of the CTD domains showed that the relative orientations of the N2 domains were largely preserved but with translational shifts, depending on the chain (Supplementary Fig. S8). To quantify these movements, we determined the centers of mass for each domain and compared shifts in distances from domains on opposite sides of each hexamer (Table 1). The CTDs for chains A and B, at the widest point of the hexamer, are further apart in the crystal structure, but the reverse is the case for chains C and D, reflecting the flatter structure of the TtPilF_c_ hexamer. The N2 domains, however, were all consistently further apart in TtPilF_c_ compared with TtPilF^AMPPNP^. Differences in the relative positions of CTD and N2 domains on each chain, which presumably also alter the conformation of the linker which contributes to ATP binding, could explain the activation of ATPase activity in the intact TtPilF hexamer, compared with the enzymatically inert TtPilF_c_.

**Table 1:** Differences in distances between centers of mass from opposing ATPase domains.

	TtPilF_c_ - TtPilF (AMPPNP) (Å)		TtPilF (AMPPNP) - TtPilF (apoprotein) (Å)	
	CTD	N2	CTD	N2
A/B	2.9^a^	8.2	6.3	7.8
C/D	−4.1	4.0	5.2	6.5
E/F	0.9	5.2	3.9	8.4

^a^Values represent distances between centers of mass for equivalent domains on opposite sides of the TtPilF hexamer. Centers of mass were calculated using CALCOM^50^. Chains are labelled in Fig. 4A.

A similar approach was used to compare the ATPase domains in TtPilF^AMPPNP^ and TtPilF^apo^. The apoprotein structure showed a consistent contraction towards the center of the complex for all domains- readily apparent from a superposition of both structures (Fig. 4B) and also an analysis of the displacement of the centers of mass (Table 1 and Fig. 4C). Displacement of the N2 domains appeared to be generally greater than the CTDs: this observation is significant because it is this domain which is linked to the N1 domains in the N-terminal half of TtPilF. It therefore appears that binding of AMPPNP to TtPilF^apo^ triggers an expansion of the hexamer, outwards from the center.

McCallum *et al*. used a comparison of structures of the PilB ATPase from *Geobacter* in AMPPNP and ADP-bound states to propose a model in which the ATPase transitions between open and closed states on binding of AMPPNP^17^. Previous work on ATPases from this family has established that the CTD and the N2 domain from the preceding chain in the hexamer form a rigid body unit, as result of the extensive interface between the two domains^17–19^. The alignment of adjacent CTD/N2 domain units can form open or closed structural states which, in turn, are dictated by occupancy of the ATP binding site. We therefore compared the two closed and one open pairings of CTD/N2 domain units from the ADP-bound form of PilB_G_ with the equivalent conformations within the TtPilF^apo^ structure. Alignment gave r.m.s. deviations of 3.5, 3.4 and 3.9 Å for the two closed and open pairings, respectively^28^. The principal reason for the deviation is that the ADP-bound form of PilB_G_ has a pronounced curvature on the N2 domain side of the hexamer, which is not the case for TtPilF^apo^. The equivalent procedure, which compared CTD/N2 pairings in closed and open states for the AMPPNP-bound forms of PilB_G_ and TtPilF^AMPPNP^, gave r.m.s. deviations of 3.1, 2.9 and 3.3 Å respectively. For comparison, superposition of the ADP- and AMPPNP-bound forms of PilB_G,_ using PHENIX^29^, gave an r.m.s. deviation of 1.4 Å. In other words, the two conformations of PilB_G_ are more similar to each other than they are to the ATPase domains of TtPilF in either state. We conclude, therefore, that the transition from TtPilF^apo^ to TtPilF^AMPPNP^ is better described by an outward shift in center of mass, particularly of the N2 domains, rather than changes in the relative orientation of adjacent CTD/N2 units. Such a difference may be attributable to the fact that electron density for the N1 domains is detected in our structures and could influence structural transitions observed within the ATPase domains. In addition, it is important to note that our cryoelectron microscopy-generated maps are not at a sufficient resolution to determine whether all three non-identical ATP binding sites are occupied by AMPPNP, or whether there are differences in catalytic competence and/or ligand binding between non-identical sites.

### Modelling of N1 domains

No crystal structures are currently available for the N1 domains from TtPilF but structures of the equivalent domains from type II secretion system ATPases are available^30,31^. Each TtPilF chain forms three N1 domains, which we will term the first, second and third, numbered from the N- to the C-terminus^25^. To dock the N1 domains into the AMPPNP density map, we first employed the segmentation facility SEGGER^32^, as implemented within Chimera^33^, to identify potential domain boundaries. The results were indicative of 12 separate domains in the lower half of the PilF hexamer i.e. two domains per TtPilF chain. It was also noteworthy that the domains formed two separate rings- an upper and a lower. We reasoned that the upper ring most likely corresponded to the third N1 domain in the TtPilF sequence, which lies adjacent to the ATPase domains. The lower ring would therefore correspond to the second N1 domain. Clearly, this implies that the density for the first N1 domain is not well resolved, possibly because it is weaker and/or unfolded to some degree. This might be because it plays a role in interaction with TtPilM (see below).

**Figure 5:**
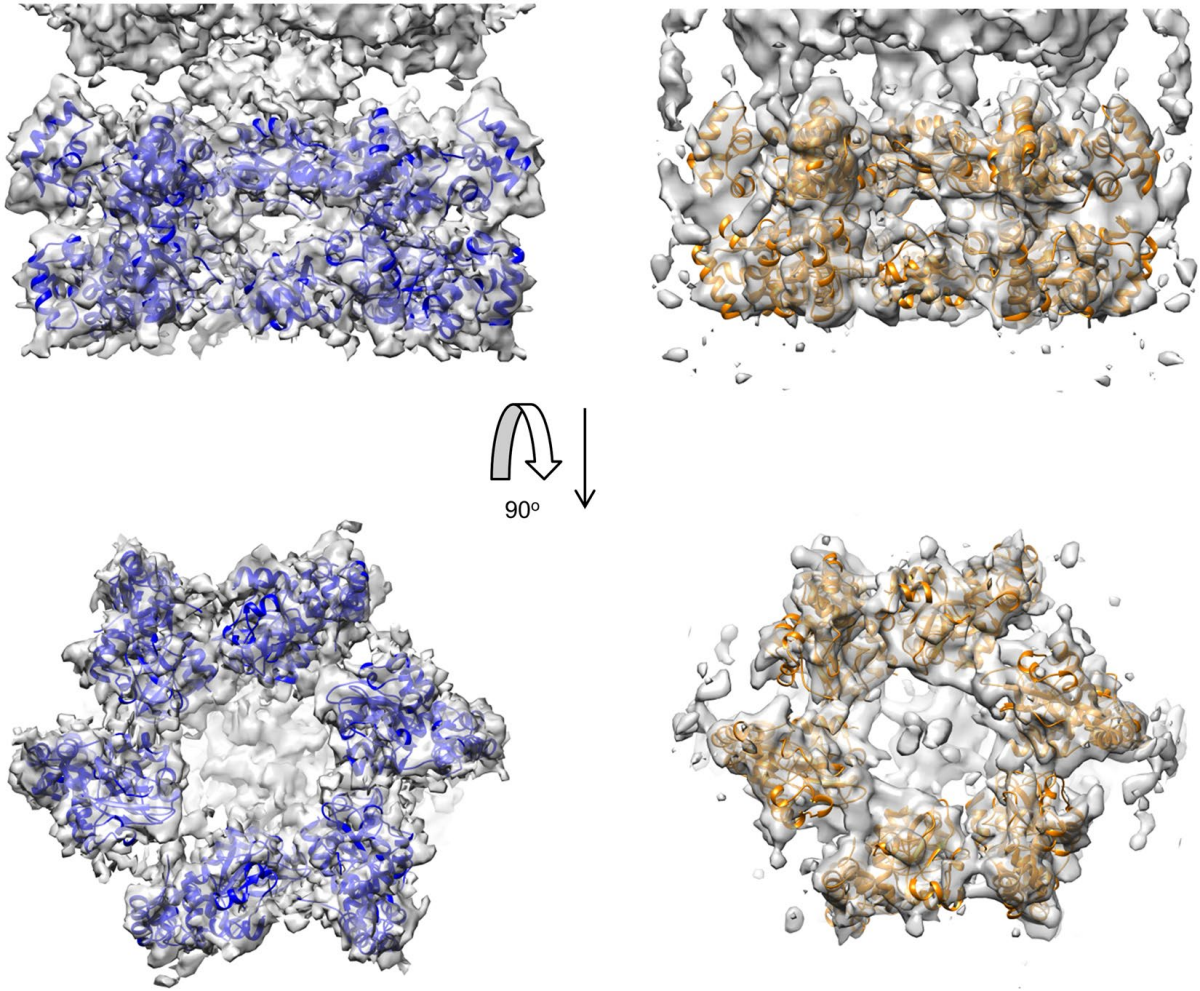
Docked conformations of N1 domains into cryoelectron density maps from the AMPPNP and apoprotein forms. Side and bottom views of N1 domain models, generated from I-TASSER, are shown docked into the AMPPNP (left) and apoprotein (right) cryoelectron density maps. The AMPPNP-bound structure is in blue and the apoprotein is in orange.

To identify the orientation of each N1 domain, models for the second and third N1 domains were generated using I-TASSER^34^, based on the structure of the MshE N-terminal domain from *Vibrio cholerae* (Supplementary Fig. S9)^31^. For each N1 domain, a comprehensive 6D search was conducted against each electron density map for the 6 separate, segmented domains. For each ring in the complete TtPilF structure, a consistent orientation of each domain was identified within the 8 highest scores for each search. The complete domains were then subject to refinement of fitting by ROSETTA^27^. This rigid body model was then transferred to the apoprotein electron density map and re-refined in ROSETTA to generate the model for the apoprotein form. Both models gave excellent agreement with experimentally determined electron density for each form (Fig. 5).

**Figure 6:**
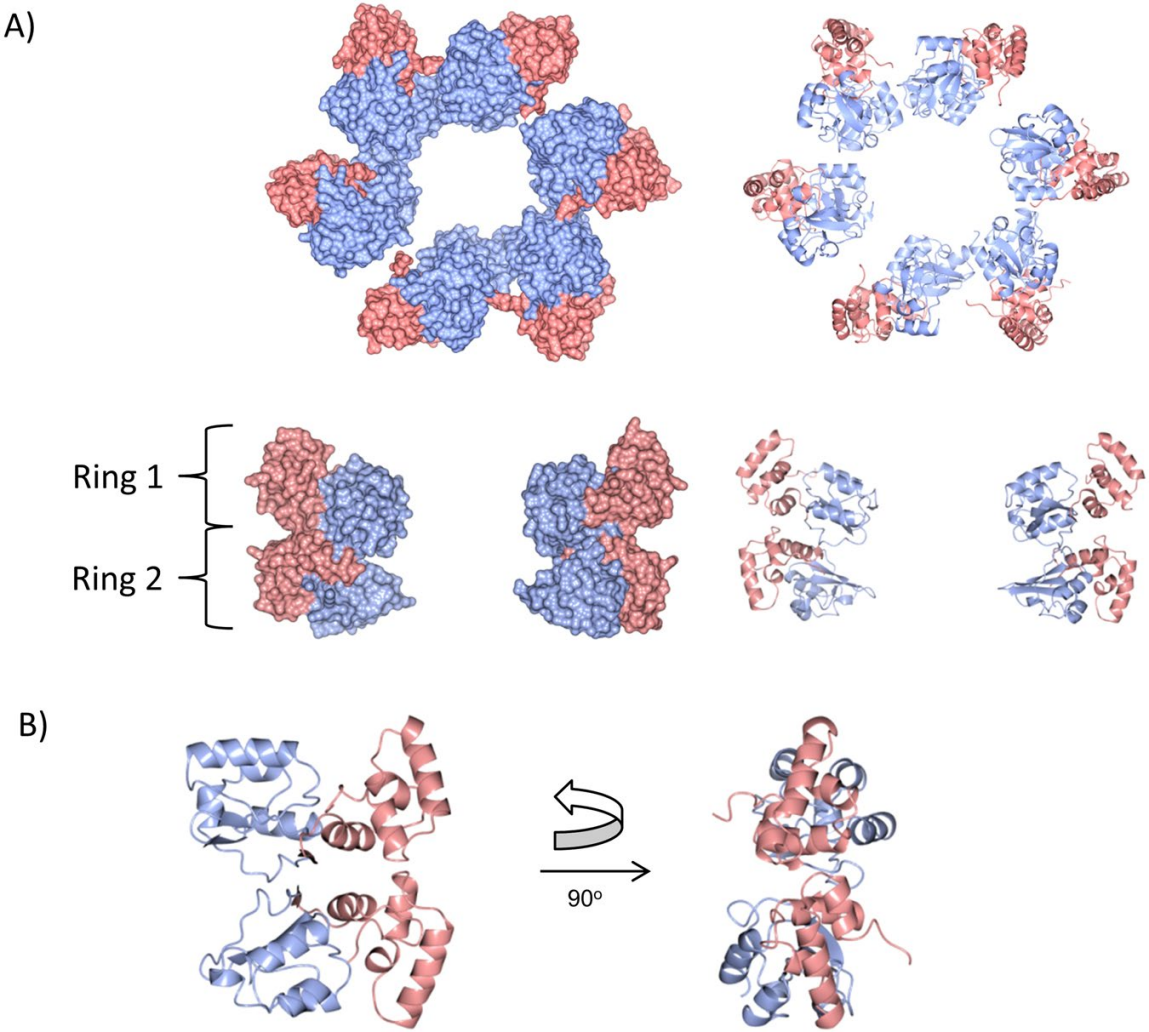
Assembly of N1 domains and sub-domains in the TtPilF^AMPPNP^ model. (**A**) Spacefilling (left) and ribbon (right) models of N1 domains from TtPilF^AMPPNP^, showing views from the bottom (upper panel) and side (lower panel). The four α-helix subdomains are in pink and the α/β subdomains in light blue. Only two subunits are shown in the lower panel, on opposite sides of the hexameric rings; this side view is orientated such that the ATPase domains lie above the N1 domains, but are also not shown. Ring 1 corresponds to the third N1 domain and ring 2 to the second N1 domain in the TtPilF sequence. (**B**) Relationship between two sample N1 domains from rings 1 and 2; the view on the left is down the quasi-two fold symmetry axis.

The MshE N-terminal domain from *Vibrio cholerae*, which forms the basis for the models of the N1 domains in TtPilF, consists of two subdomains: a four α-helix bundle and a 3-stranded β-sheet packed against 3 additional helices^31^. Collectively, the N1 domains stack into a cylinder, with external dimensions of about 130 Å wide by 55 Å in height. The model of the TtPilF cylinder is formed from two rings, each containing 6 N1 domains; the N1 domains are orientated such that the four α-helix subdomains lie on the outside of the cylinder (Fig. 6A). The α/β subdomains line the core, which forms a channel approximately 40 Å in internal diameter. In the model, the docked N1 domains in the upper and lower rings are proposed to be related by quasi-two fold symmetry of the four helix subdomains, with the symmetry axis perpendicular to the main C2 symmetry axis of the complex. The major site of interaction between adjacent N1 domains in the upper and lower rings is the fourth helix in the four α-helix sub-domain and the linker peptide which connects the two domains (Fig. 6B).

### Movement of N1 domains in response to structural changes in the ATPase domains

**Figure 7:**
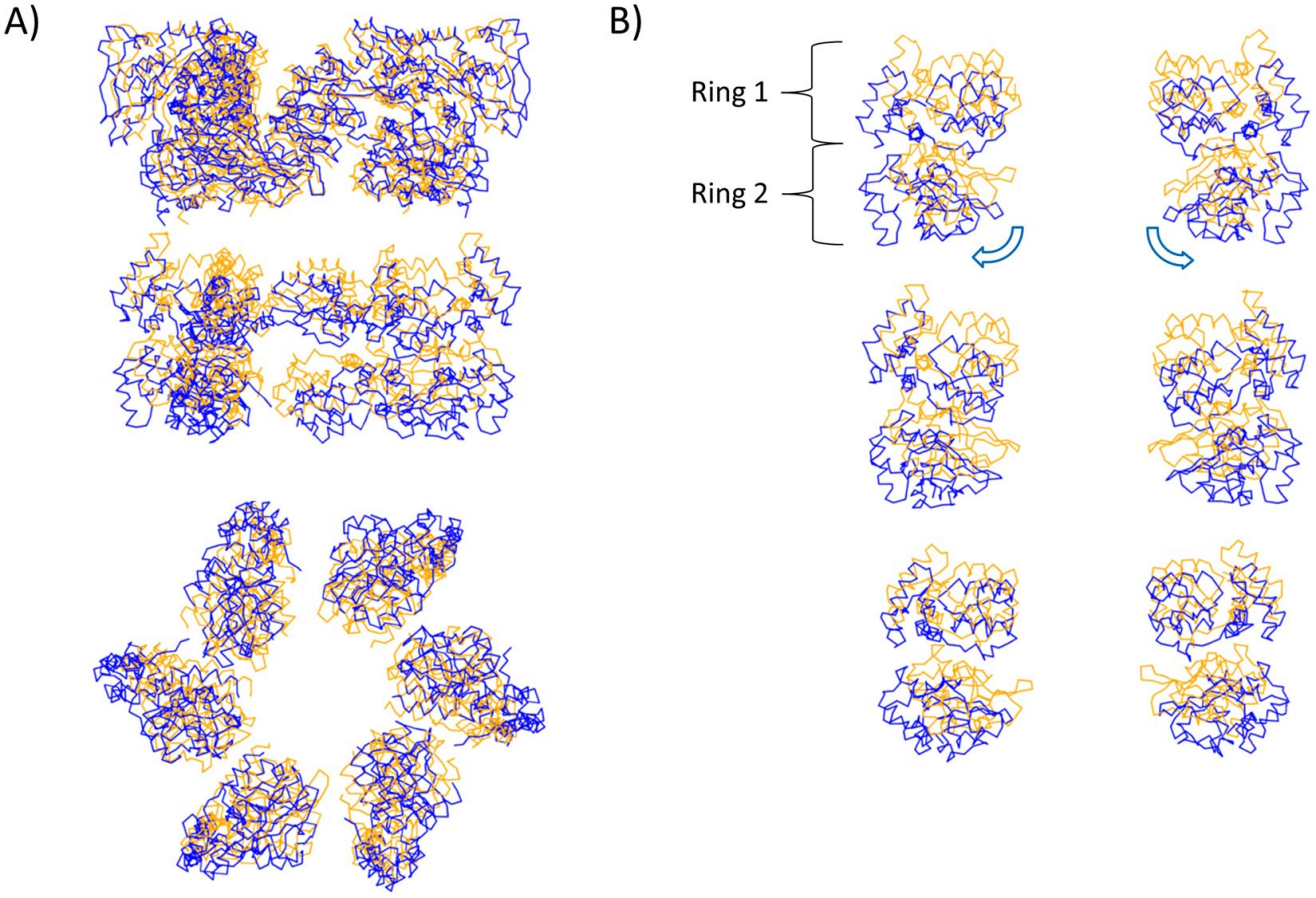
Comparison of structures of N1 domains from TtPilF^AMPPNP^ and TtPilF^apo^. The ATPase domains in TtPilF^AMPPNP^ were superimposed onto those from TtPilF^apo^. (**A**) Side and bottom view of the N1 domains from TtPilF^AMPPNP^ (blue) and TtPilF^apo^ (orange). (**B**) N1 domains as pairs of subunits on opposite sides of the hexamer. Arrows indicate the direction of rotation outwards.

In order to examine the motion of the N1 domains relative to the ATPase domains in TtPilF^AMPPNP^ and TtPilF^apo^, the ATPase domains from TtPilF^AMPPNP^ were superimposed onto their equivalents from TtPilF^apo^ ^28^. Calculations of centers of mass for each domain showed that the TtPilF^AMPPNP^ N1 domains from both ring 1 and ring 2 are displaced downwards by distances of 4–9 Å (Fig. 7). This is accompanied by rotation outwards (mean value 14^o^ for ring 2 domains), which leads to a wider aperture at the base of the structure. Combined, these movements lead to changes in the relative positions of the N1 domains in ring 2 of 10–13 Å which occur on the transition from TtPilF^AMPPNP^ to TtPilF^apo^ and therefore, by implication, after hydrolysis of ATP. Conversely, this means that the N1 and ATPase domains are drawn closer together in the apoprotein state. It seems likely that the movement of the N2 domains plays a critical role in this structural transition although, at this resolution, we were unable to directly trace the path of the polypeptide chain from each N2 domain to its N1 counterpart. The precise way in which the relative movement of the CTD and N2 domains is transmitted to the N1 domains in ring 1 therefore remains unclear. However, we note that the central mass of density which lies on the symmetry axis, which we termed the ‘stem’ in our previous work^25^, is not accounted for in our models and could be attributed to the missing section of the model between the N1 and N2 domains. This would confirm our previous hypothesis- that this central part of the TtPilF hexamer plays a critical role in linking structural transitions in the ATPase domains which occur on binding and hydrolysis of ATP with displacement of the N1 domains in the N-terminal region of the molecule.

## Discussion

ATPases play a critical role in powering both TFP biogenesis and type II secretion, but the precise manner by which ATP hydrolysis is coupled to secretion and fiber assembly is still unclear. They are members of the much larger family of AAA+ ATPases which are involved, not just in secretion, but also a range of other cellular processes, such as chaperone-related and protein folding functions^14^. Phylogenetic analysis shows that the PilB/GspE subgroup, which is associated with type IVa pilus biogenesis and type II secretion, forms a distinct clade separate from the BfpF and the PilT/PilU clades^17^. Structural studies on the T2SS ATPase GspE have been complicated by difficulties in obtaining an assembled hexamer, requiring engineering of the assembly into a hexameric state^19^. By contrast, *T. thermophilus* PilF can be isolated as an intact hexamer and is somewhat unusual, in that it harbours 3 predicted N1 domains in the N-terminal half of the molecule. However, we found density for only 12 such domains, leading us to conclude that electron density for the first N1 domains from each of the 6 chains in the complex is weak in our cryoelectron microscopy maps. Recently Kruse *et al*. showed that expression of TtPilF in which GSPIIA and GSPIIB, or all 3 GSPII/N1 domains, disrupted hexamer assembly when removed^35^. This is consistent with our structural model, which shows contacts between adjacent GSPII/N1 domains in the hexamer- disruption of these contacts could destabilise assembly. The GSPII/N1 domains were found to be not required for ATPase activity but, interestingly, mutation of the GSPII domains had effects on transformation efficiency and twitching motility^35^.

Addition of pilin subunits from the inner membrane into a growing or retracting pilus fiber would require sequential insertion or removal from the fiber base in a clockwise or counter-clockwise manner. Models for ATPases which drive assembly or disassembly therefore need to incorporate an explanation of how this phenomenon is linked to ATP hydrolysis. Nivaskumar *et al*. analyzed pilus fibers formed from the type II secretion pilin PulG, finding experimental evidence for variation in the twist angle, which was confirmed by computation, leading to a three stage pilus assembly model^7^. The first assembly stage is accompanied by a displacement of 10.4 Å, which removes the pilin from the inner membrane in a spooling-type mechanism. Interestingly, this displacement value is similar to the shift of the N1 domains relative to the ATPase domains which we observe on binding of AMPPNP. We find that TtPilF_c_ is wider and flatter than its equivalent density in the complete hexamer in the AMPPNP-bound state, indicating that the presence of the N1 domains does affect the relative orientations of the ATPase domains. McCallum *et al*. compared the structures of the *Geobacter metallireducens* PilB ATPase in AMPPNP and ADP-bound states, leading to a model in which pairs of protomers hydrolyse ATP in a sequential clockwise direction^17^. However, neither of the PilB structures had clear electron density for the N1 domains, which could influence the conformational states of the ATP-binding domains within the complex. The authors also reinterpreted structures of the PilT retraction ATPase, originally published by Satyshur *et al*.^20^, according to the same model which suggested that PilT employs a similar mechanism, but operating in the reverse direction, thus disassembling a right-handed pilus helix. Despite the lower resolutions obtained, an advantage of the single particle approach is that it allows a consideration of the complete TtPilF structure and thus avoids potential concerns about crystal packing artefacts.

Our observations on TtPilF structures provide little indication of a rotary mechanism. Binding of AMPPNP is accompanied by an outward expansion of the CTD and N2 domains which is linked to a downward and outwards displacement of N1 domains on binding of AMPPNP (Fig. 7). A key question is, how might this be related to displacement of a pilin subunit out of the inner membrane? A displacement-type mechanism would require anchoring of TtPilF to the inner membrane, or components within it. Current evidence, from other TFP biogenesis systems, suggests that PilF interacts with the inner membrane protein PilC and the PilMNO complex, which could provide the anchoring necessary. Formation of such a complex could provide the environment for engagement with a membrane-bound pilin, reducing the activation energy necessary to guide it out and into the nascent pilus fiber. Possibly the first N-terminal N1 domains, which we do not observe in our structures, could play a key role here, by engagement with inner membrane components.

Although there is no complete structure of a PilB-type ATPase with defined N1 domains yet reported, a complex of GspE bound to GspL- an inner membrane component of the T2SS- has been published^36^. Here the CTD and N2 domains adopt an unusual orientation such that, when compared with their counterparts from TtPilF, the N2 domains lie on different sides of the CTD (Supplementary Fig. S10). The N1 domain, as well as GspL, lies close to the CTD in GspE. This is a radically different arrangement from TtPilF, where there is relatively little contact between the CTD and N2 domains on the one hand and the N1 domains on the other in the TtPilF complex- they are apparently held together by the stem structure discussed earlier. However, GspE in the *Vibrio* complex does not form a hexamer in the crystalline state, which could contribute to differences in the relative locations of the constituent ATPase domains.

Recent results obtained by cryoelectron tomography have provided valuable insights into the TFP biogenesis machine *in vivo*^21–23^. Of particular relevance here is the study by Gold *et al*., which determined the structures of the TFP machinery in open and closed states^21^. Although the PilQ secretin and other periplasmic components are well resolved in the tomograms, the parts of the assembly platform in the inner membrane and cytoplasm are less clear. This is also the case for the tomograms from *Myxococcus*^22^ and *Vibrio*^23^ and has been taken as an indication of a dynamic assembly platform at the cytoplasmic surface of the inner membrane^37^. Clearly there is a need for a dynamic interchange between PilB-type assembly ATPases and retractile PilT ATPases; contacts with the inner membrane components of the machine will need to be exchangeable. PilB (or TtPilF) is thought to operate through binding to the PilC integral membrane protein^38^ and PilM, which forms a complex with PilN and PilO^10^. Further work will be required to determine how structural changes in TtPilF are communicated to PilC and PilMNO, and what role these other components play in mediating type IVa pilus fiber assembly.

## Methods

### Expression and purification of PilF and PilF_c_

The full length *Thermus thermophilus* HB8 *pilF* gene was amplified from genomic DNA using PCR and cloned into the pET15b vector as described previously^25^. The final construct contained a 6×-His tag followed by thrombin cleavage site at the N-terminus of PilF. The plasmid was transformed into T7 express cells (New England Biolabs) and few colonies were used to inoculate a 100 mL of 2×-YT media containing ampicillin (100 μg/ml) and grown at 37 °C for 3 hours. The starter culture was diluted into 4 L of fresh 2×-YT media and the cells were allowed to grow at 37 °C until the OD at 600 nm reached 0.6–0.8. At this stage, the culture was cooled to 16 °C and IPTG was added to a final concentration of 0.1 mM and allowed to grow for 16 hours. The cells were harvested by centrifugation at 6000 g for 10 min, resuspended in lysis buffer (25 mM HEPES pH 7.5, 150 mM NaCl, 5 mM MgCl_2_ and 5% glycerol (v/v)) and lysed by sonication. The debris was removed by centrifugation at 30,000 g for 30 min, the supernatant was passed through a 0.45 μm filter and followed by a 0.2 μm filter. The filtered solution was injected into a 5 ml HisTrap column (GE Healthcare) using a peristaltic pump. The column was washed with lysis buffer containing 20 to 100 mM imidazole pH 7.5 by step gradients of 3 column volumes each. Increasing the imidazole concentration to 500 mM eluted the PilF protein. The protein was incubated at 50 °C for 15 min and then centrifuged at 18000 g for 15 min and the supernatant was collected.

For cryoelectron microscopy studies, the full length PilF from the previous step was concentrated and injected into a Hiload Superdex 200 (16/60) column (GE Healthcare) equilibrated with lysis buffer. The pure fractions from the elution were pooled and concentrated to 1 mg/ml. To form the PilF-AMPPNP complex, AMPPNP was added to a final concentration of 1 mM.

For crystallization studies, the full length PilF from the metal chelate affinity and heating steps was concentrated to ~4 mL, and then ATP was added to a final concentration of 2 mM. To this, trypsin was added (PilF:trypsin ratio of 1:100 (w/w)) and incubated at 37 °C for 30 min. Powdered complete protease inhibitor tablet (Roche) was added to the sample to stop the trypsin digestion and then injected into a Hiload Superdex 200 (16/60) column equilibrated with lysis buffer supplemented with 2 mM ATP. The pure fractions from the elution were pooled and concentrated to 16 mg/ml. Analysis by SDS-PAGE and mass spectrometry revealed almost all of the N-terminal GSPII domains were digested by the trypsin treatment.

For selenomethionine (SeMet) labeling, the cells were grown in SeMet media (Molecular Dimensions Ltd) as per the manufacture’s guidelines. The cells were lysed and the protein was purified as explained for the native protein except that the lysis buffer was supplemented with 1 mM DTT throughout.

### ATPase assay

The ATPase activity was measured using a phosphate assay kit (Abcam), following the manufacturer’s guidelines. Samples (200 µl) containing TtPilF (30 µg) were mixed with ATP (55 µM) and incubated at 55 ^o^C for 15 min. The phosphate reagent (30 µl) from the kit was then added to the samples and incubated for 30 min at room temperature, protected from light. Optical density at 650 nm was measured for samples using a microplate reader (Biotek). Experiments were carried out in triplicate.

### Crystallization and crystal structure determination

PilF_c_ was screened against eight commercial crystallization screens (Molecular Dimension and Microlytic) using the MRC 2-well crystallization plates and Mosquito robot (TTP labtech). The crystallization plates were incubated at 20 °C and plate-like crystals appeared within one week. PilF_c_ crystals belonged to C2 space group and grew in 0.2 M sodium bromide, 0.1 M Bis-Tris propane pH 6.5, 20% (w/v) PEG 3350. The presence of a translation non-crystallography symmetry, combined with diffraction anisotropy in one direction complicated the identification of an unambiguous molecular replacement (MR). During this time, structures of the T2SS ATPases GspE and EpsE and the T4P retraction ATPase PilT were the only available search models for MR. The optimal quality dataset was obtained from a crystal treated with K_2_PtCl_4_, as part of attempts to extract phase information.

Our efforts to obtain experimental phasing information by collecting data on native crystals soaked with heavy atoms were only partially effective: SeMet incorporated protein was therefore purified and crystallized. Although the SeMet crystals diffracted only to 4.0 Å resolution, an unambiguous solution was found by combining SAD and MR with PilT coordinates (PDB 2GSZ, three chains) as the search model in Phaser^39^. This weak solution was then extended to the higher resolution dataset (crystal treated with K_2_PtCN_4_), which also belonged to the same crystal form as the SeMet dataset. The models were built using the Autobuild wizard^40^ as incorporated in Phenix^29^. The model was completed by iterative rounds of manual building in Coot^41^ and refinement using phenix.refine^42^. The model was optimized using PDB_REDO^43^ and validated using molprobity tools^44^. We did not observe any conformational changes between the low resolution SeMet dataset and the high resolution dataset. The data and refinement statistics are presented in Supplementary Table 1 and a Ramachandran plot is given in Supplementary Fig. S11.

### Cryoelectron microscopy

3 μl of samples at a concentration of ~200 μg/ml where absorbed to freshly glow discharged gold 200 mesh Quantifoil R2/2 grids using an FEI Vitrobot. After a brief incubation at 90% humidity, grids were blotted continuously for 4–6 seconds and plunge frozen in liquid ethane. Data were acquired on an FEI Titan Krios at the electron Bio-Imaging Centre at Diamond Light Source. Movies were acquired in counting mode on a Gatan GIF Quantum LS at 1.38 Å/pixel and a 20 eV energy filter was used. The dose rate for movie acquisition was 5e/pixel/s and a total dose of ~40 e/Å2 was accumulated over 32 movie frames. Individual frames were corrected for drift and motion then summed with dose weighting using MotionCorr^45^. Approximately 40,000 clear particles were semi-automatically picked using E2Boxer and initial reference free class averages were generated in EMAN^46^ and independently cross-validated using RELION^47^. Bad particles and contaminated classes were removed and subsequent class averaging clearly identified a hexameric particle with a distinctive C2 oligomeric arrangement and therefore a low resolution start model was generated with this symmetry applied. 6 rounds of iterative refinement were performed to produce 3D volumes.

### Structure refinement into electron density

Refinement was carried out using the ROSETTA relax protocol^27^, with constraints against the electron density map, but without B-factor refinement. 100 separate simulations with either the ATPase domains or combined N1 domains were carried out against electron density maps of either the apoprotein or AMPPNP-bound forms. The lowest energy models were selected in each case. Figures were generated using Chimera^33^ and CCP4MG^48^.

## Electronic supplementary material

Supplementary Information

## Data Availability

Coordinates and structure factors for the TtPilF_c_ crystal structure were deposited with accession code 5OIU. Electron density maps and fitted models were deposited with the following accession numbers: AMPPNP-bound form, EMD-4194, PDB 6F8L; apoprotein form, EMD-3882, PDB 6EJF. Other data are available in the article and associated Supplementary Information Files.
