# Supplementary material for: Structural cycle of the Thermus thermophilus PilF ATPase: the powering of type IVa pilus assembly
Source: Sci Rep. 2018 Sep 19;8:14022. doi: 10.1038/s41598-018-32218-3 (PMC6145873; doi:10.1038/s41598-018-32218-3)
Supplement: Supplementary file 1 — Supplementary Information [file 41598_2018_32218_MOESM1_ESM.pdf]

Structural cycle of the *Thermus thermophilus* PilF ATPase: the powering of type IVa pilus assembly

Richard Collins, Vijaykumar Karuppiah, C. Alistair Siebert, Rana Dajani, Angela Thistlethwaite and Jeremy P. Derrick

Table S1

Data collection and refinement statistics

Crystal	SeMet	Pt
Space group	*C2*	
Unit cell parameters	221.9, 106.7,140.1Å; β=113.9^{o}	220.4, 107.7, 137.8Å; β=113.0^{o}
X-ray source and wavelength (Å)	DLS[b] IO4 (0.979)	DLS I03 (1.072)
Resolution range (Å)	43 – 4.00 (4.28-4.00)[a]	95 – 2.44 (2.50-2.44)
Multiplicity	10.1 (10.6)	4.5 (3.2)
Significance (<I>/sd)	9.7 (6.9)	11.5 (2.0)
No. unique reflections	25,491	109,033
Completeness (%)	99.9 (100)	98.8 (88.9)
R_{merge} (%)[c]	19.9 (35.5)	10.4 (53.4)
**Refinement Statistics**		
R_{cryst}	-	19.0
R_{free}	-	24.6
Nonhydrogen atoms		
All		18,464
Water		379
Mean overall B (Å^2)		48.0
Wilson B (Å^2)		41.81
RMSD from ideal values		
Bond distance (Å)		0.009
Bond angle (degrees)		1.05
Ramachandran plot		
		Outliers: 0%
		Allowed: 1.52%
		Favored: 98.48%

[a] values in parentheses refer to the outer resolution shell
[b]Diamond Light Source
[c]$R_{merge} = \Sigma_{hkl}\Sigma_{sym}|I-\langle I\rangle|/\Sigma_{hkl}I$

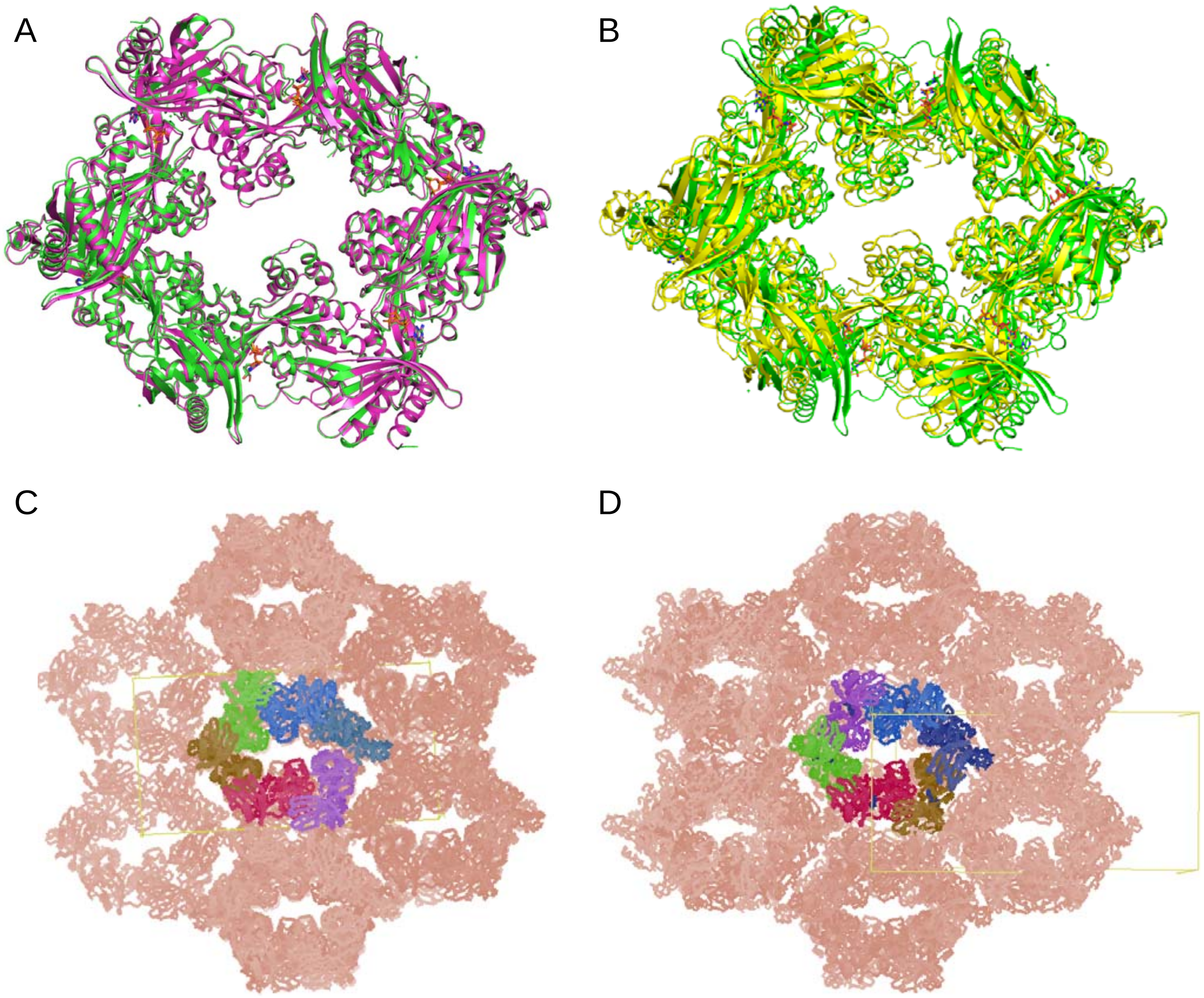

Figure S1 Comparison of $PilF_c$ with other type IV pilus assembly ATPases. A) Overlay of $PilF_c$ (green; this paper) with $PilF_c$ structure from *Thermus thermophilus* HB8 (PDB 5IT5, magenta) B) Overlay of $PilF_c$ (green; this paper) with PilB from *Geobacter metallireducens* (PDB 5TSH, yellow) C). Crystal packing of $PilF_c$ (this paper) with unit cell. D) Crystal packing of $PilF_c$ from *Thermus thermophilus* HB8 with unit cell (PDB 5IT5).

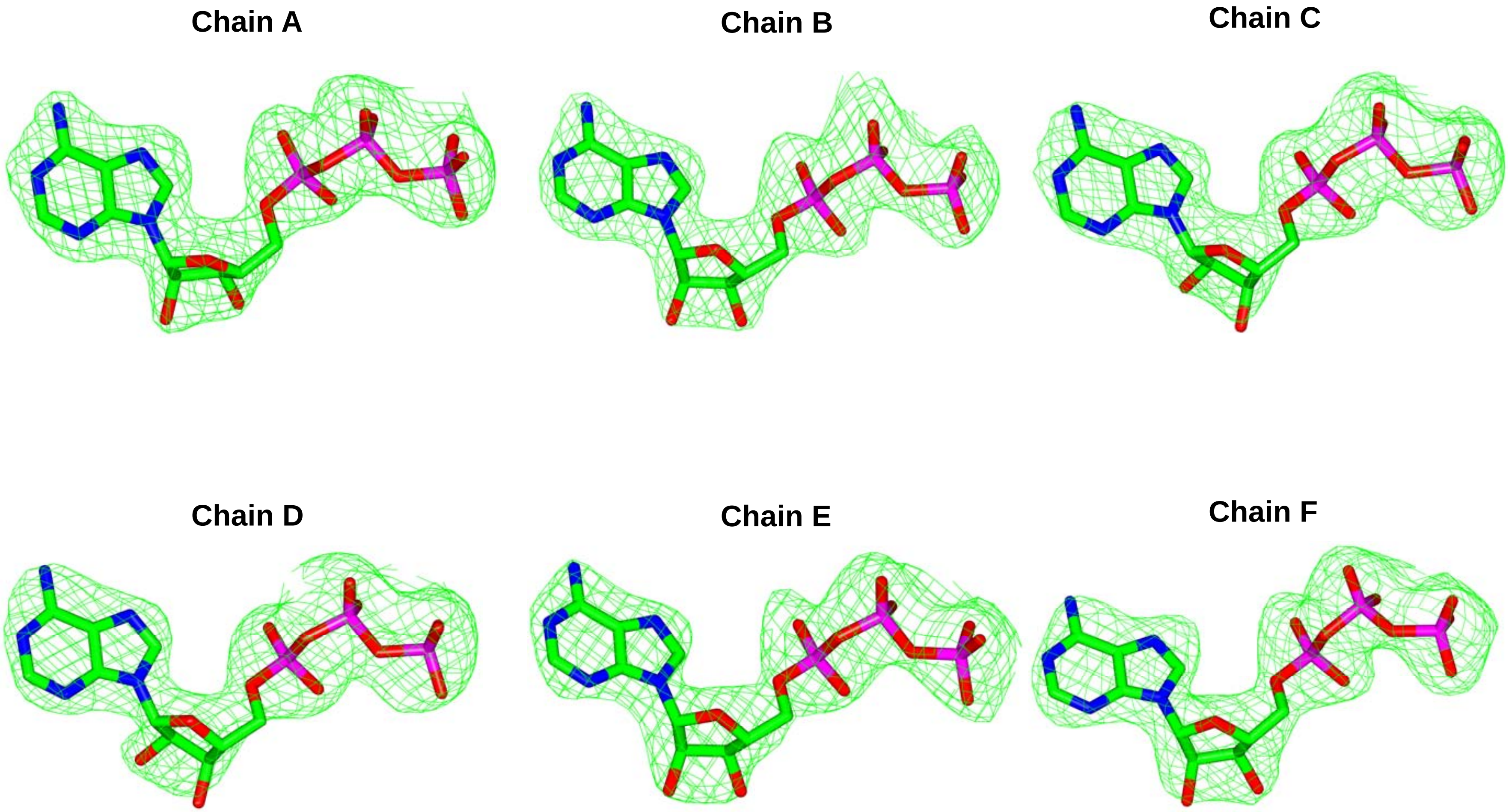

Figure S2 (Fo-Fc) difference map (green mesh) of ATP molecules in six different chains of TtPilFc. The map was contoured at 3σ. The atoms in ATP (sticks) are shown as follows: carbon in green, nitrogen in blue, oxygen in red and phosphorous in magenta.

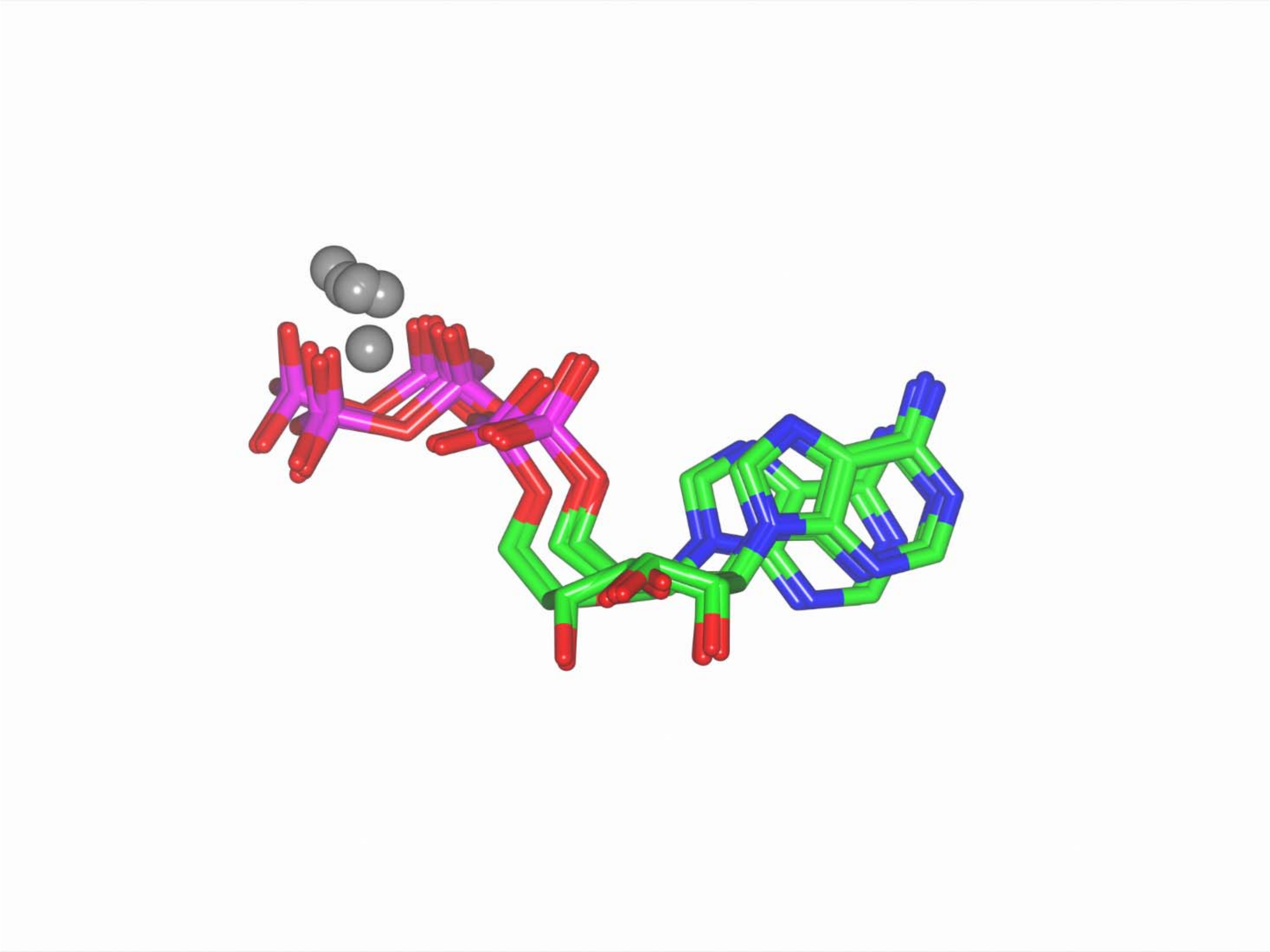

Figure S3 Superposition of ATP/Mg^{2+} from all 6 chains in TtPilFc

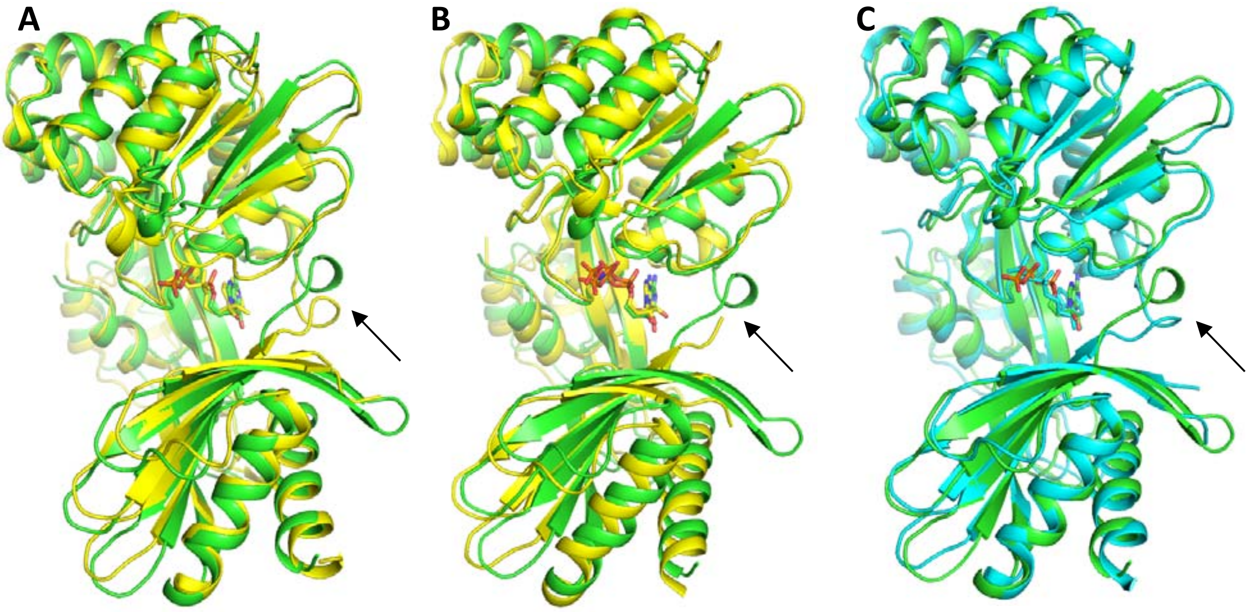

Figure S4 Comparison of the conformation of linker polypeptides between PilF$_c$ and *Geobacter metallireducens* PilB. A) Overlay of PilF$_c$ (chain A, green) and PilB (chain F, yellow) from *Geobacter metallireducens* (PDB 5TSH) bound to ADP (crystallised with AMP-PNP). B) Overlay of PilF$_c$ and PilB (chain A, PDB 5TSH) bound to AMP-PNP. C) Overlay of PilF$_c$ and PilB (chain B, cyan) bound to ADP (PDB 5TSG). Arrows denote the linker regions.

A

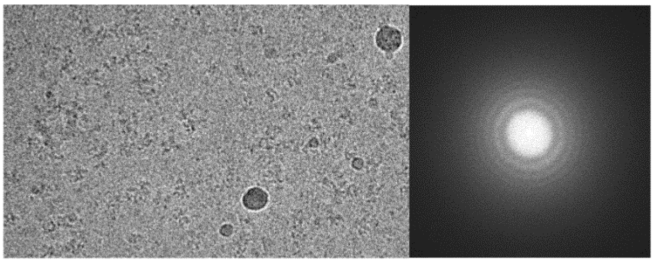

B

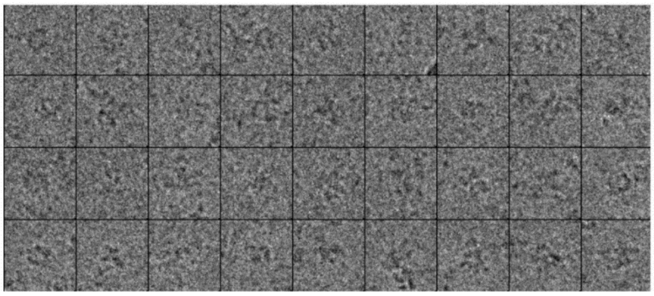

C

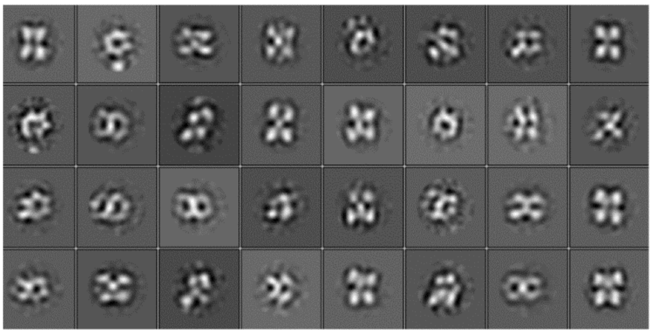

D

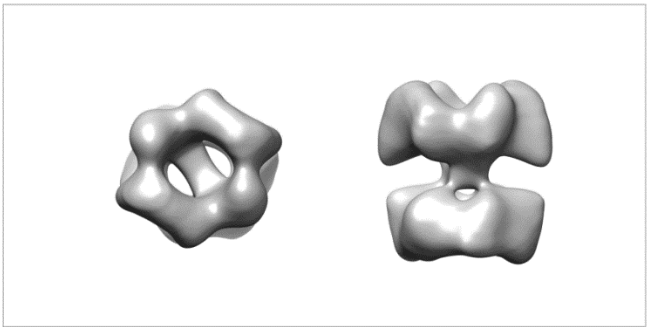

Figure S5 Examples and processing of the TtPilF (AMPPNP-bound form) cryoelectron data. A) Field of view of an area of TtPilF in thin vitreous ice. Thon rings extended past 6Å. B) Examples of individual non-overlapping TtPilF complexes selected from the area shown in A). Box size = 216Å   C) Initial class averages generated from a small dataset of 2500 particles. Data were low-pass filtered to 20Å resolution to prevent any reinforcement of noise. D) An initial C2 start model generated from the classes shown in C) and used to independently refine to high resolution.

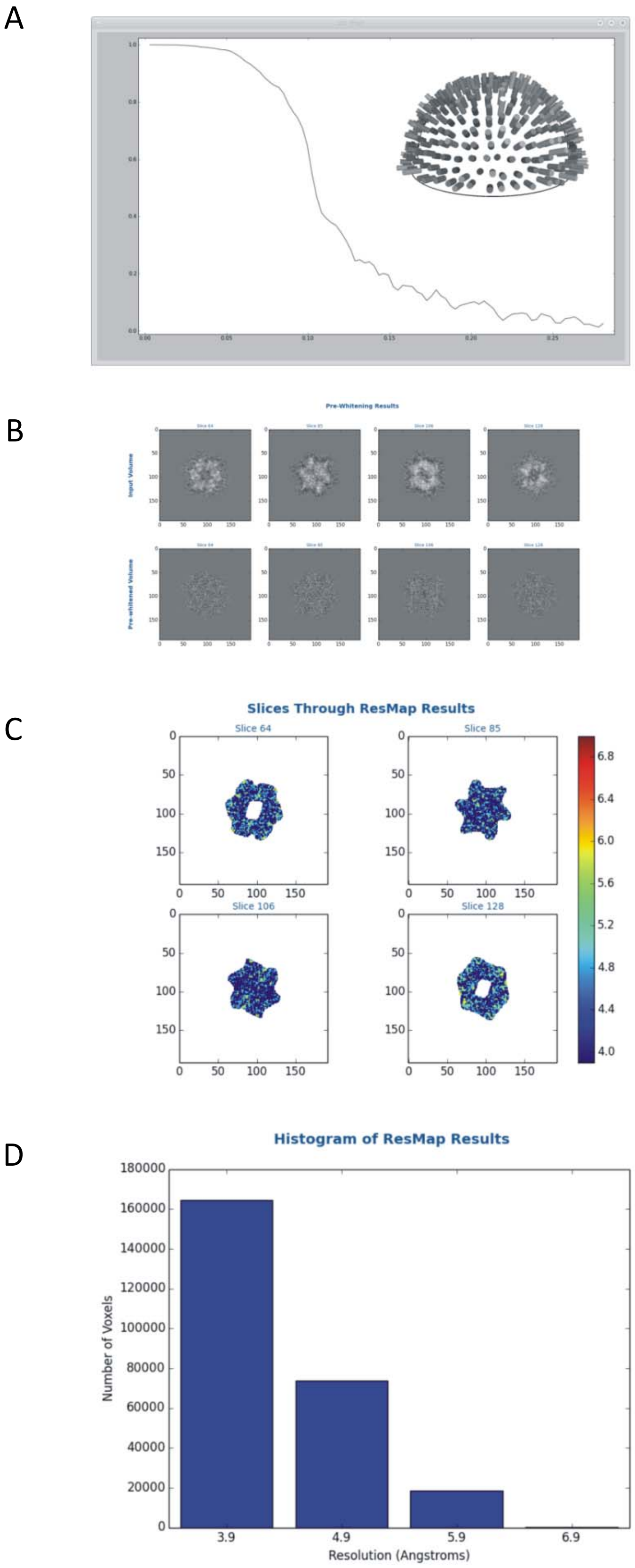

Figure S6 Resolution estimation of TtPilF (AMPPNP-bound form) A) FSC calculation of the C2 refinement. At the conservative value of 0.5, a resolution of 8Å is recorded. The inset box shows the projection sampling of C2 Euler space and indicates no gaps in coverage. B) RESMAP PW results for the TtPilF volume, displayed as slices parallel to the long axis through from the ATPase to the N1 domains. C) RESMAP Resolution variation through the height complex shows a uniform distribution. D) Histogram of resolution difference.

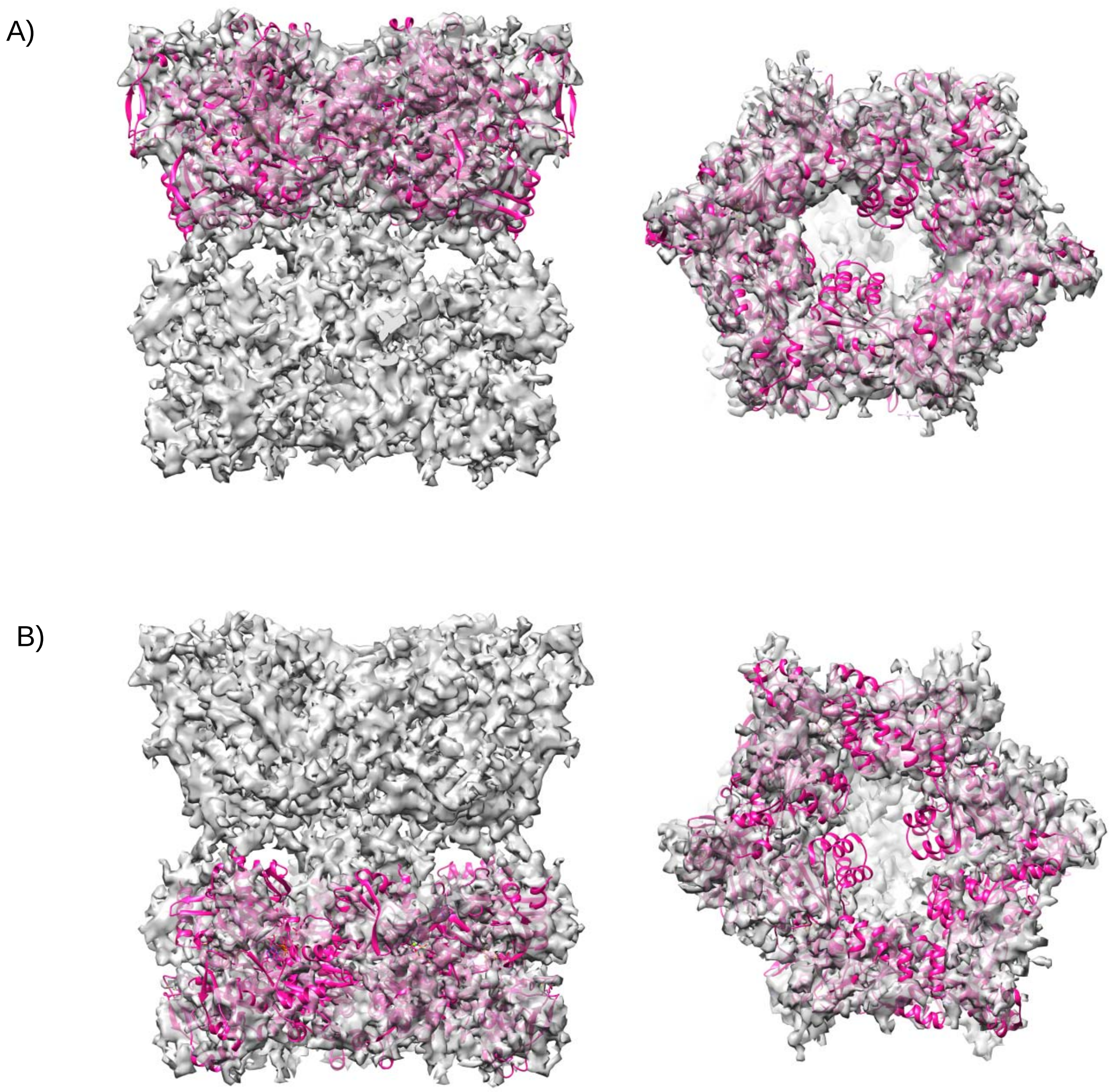

Figure S7 Alternative docking orientations of the TtPilFc crystal structure into the cryoelectron microscopy density map (AMPPNP-bound form) A) Side and top views of the correctly orientated structure (correlation = 0.83) B) Side and top views of PilFc docked into the opposite density mass (correlation = 0.70). Rigid body fitting was carried out in Chimera [33].

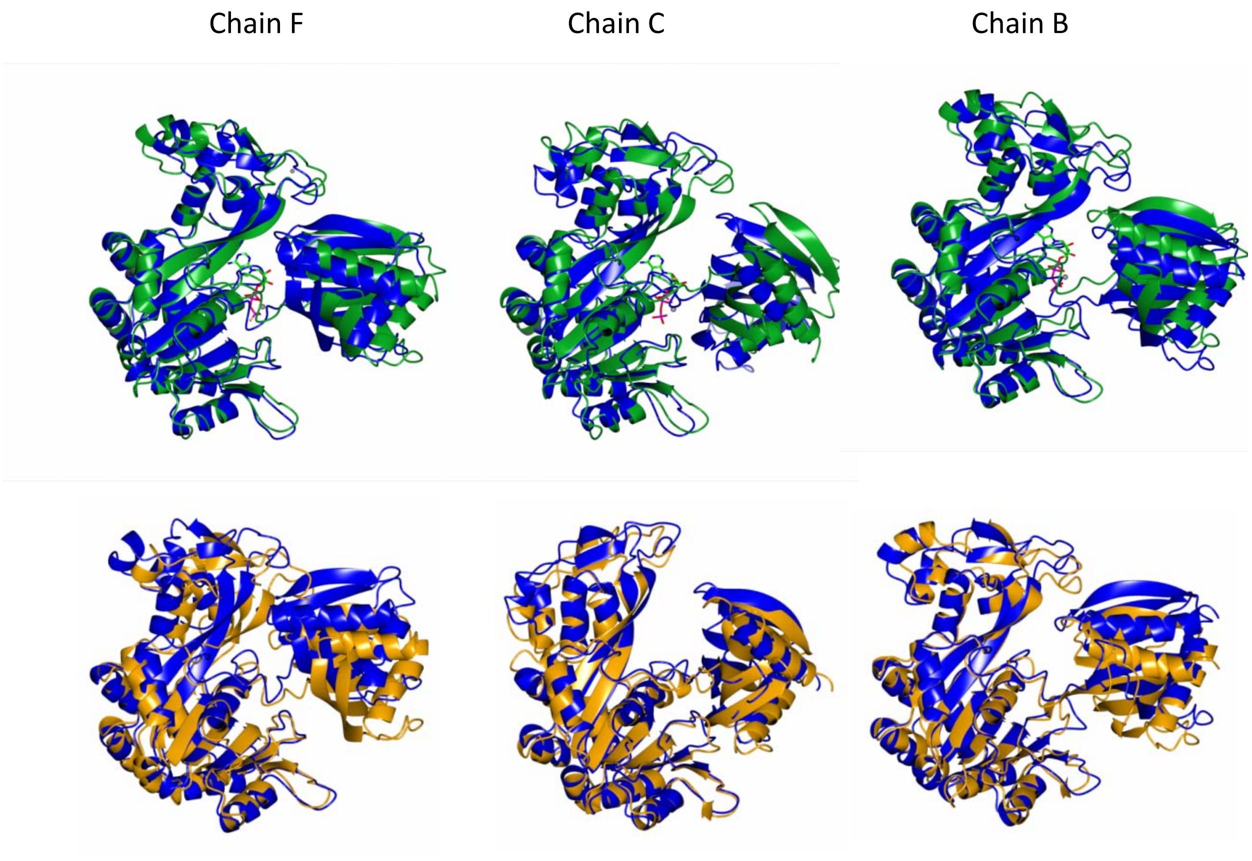

Figure S8 Comparison of conformations of CTD and N2 domains for chains F, C and B. Cα atoms from each CTD domain were superimposed by least squares fitting. Top panel: $PilF_c$ (green) and $TtPilF^{AMPPNP}$ (blue). Bottom panel: $TtPilF^{AMPPNP}$ (blue) and $TtPilF^{apo}$ (yellow).

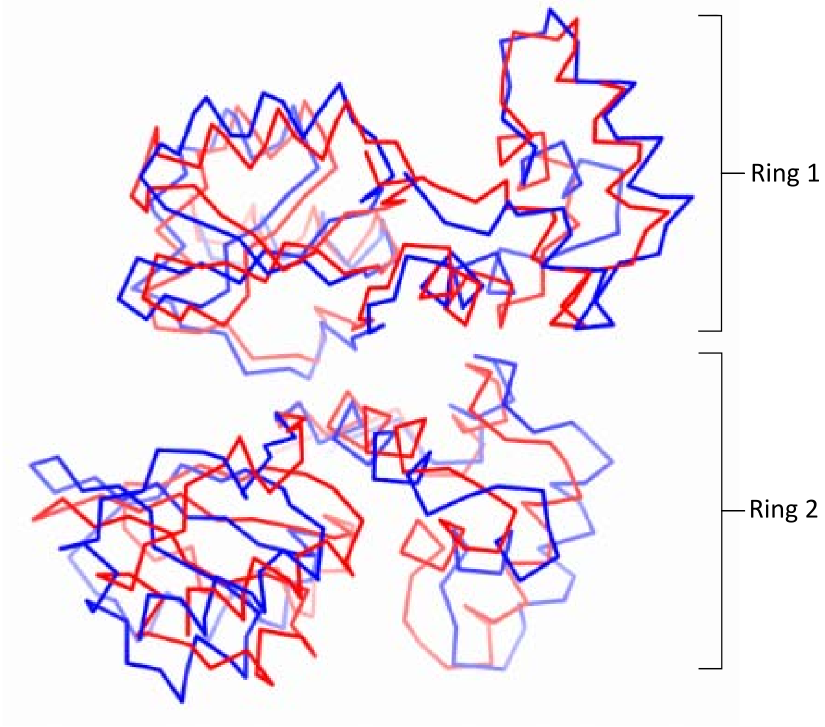

Figure S9 Comparison of TtPilF GSPII domains (AMPPNP-bound form) with *V. cholerae* MsnEN domain . Superposition of GSPII domains from TtPilF (red) with the MsnEN template structure from *V. cholerae* (blue; PDB 5HTL). Structural overlay was carried out using CCP4MG [48]; rmsd values were 2.3Å (ring 1) and 3.4Å (ring 2).

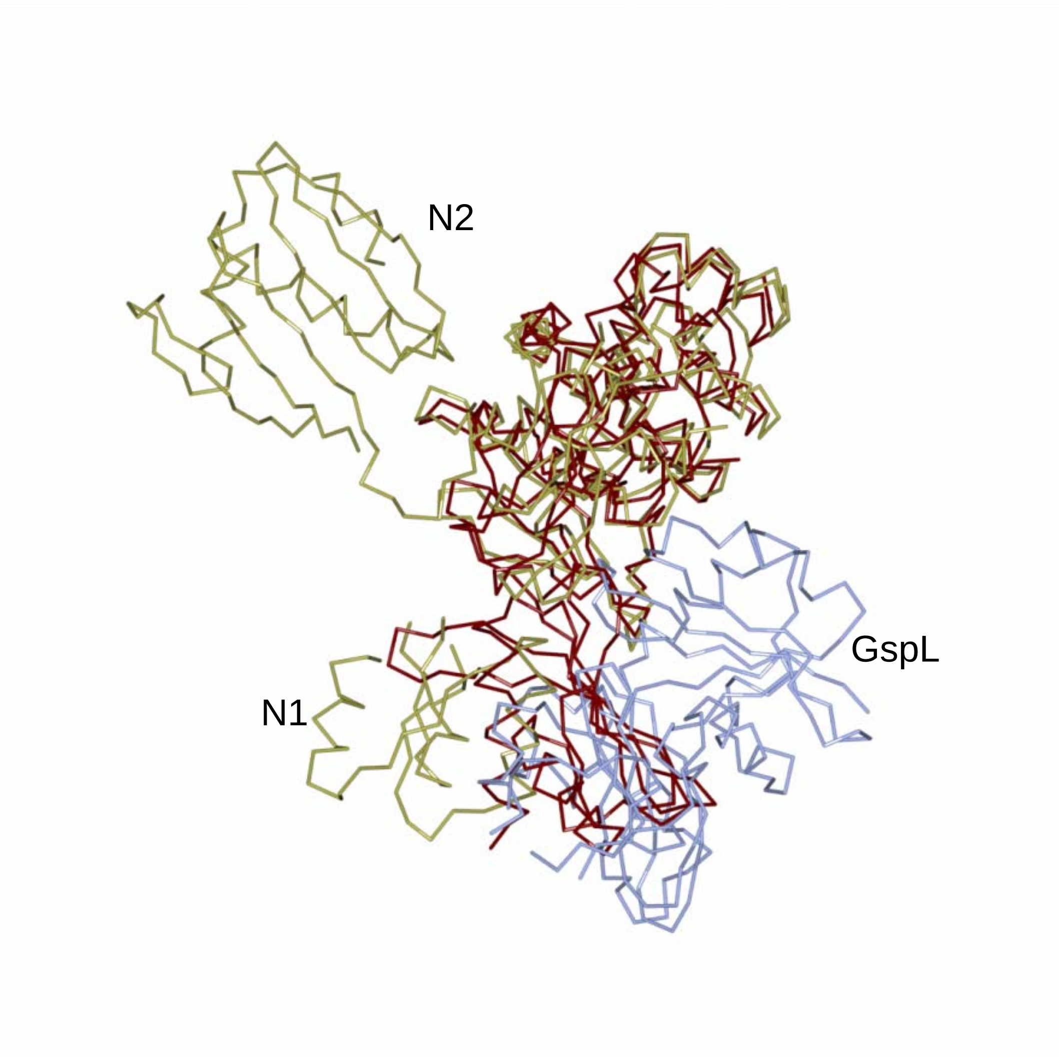

Figure S10 Comparison of TtPilF ATPase domain with the GspE-GspL complex from *Vibrio vulnificus*. CTD domains from TtPilF (chain A, AMPPNP-bound form) and GspE (PDB code 4PHT) were superimposed (rmsd 1.84Å). TtPilF is shown in brown, GspE in gold and GspL in light blue. The relevant domains from GspE are labelled.

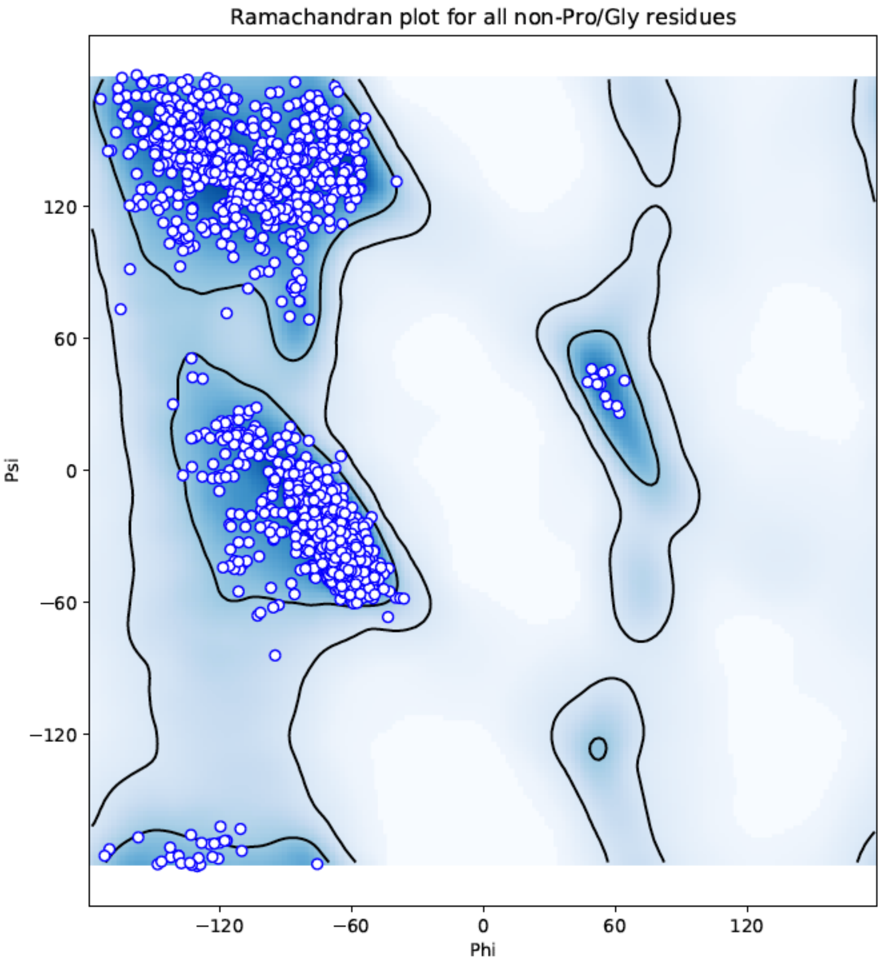

Figure S11 Ramachandran plot for TtPilF$_c$ crystal structure
